# Nyquist Sampling Conditions of Some Diffraction Algorithms with Adjustable Magnification

**DOI:** 10.3390/s23031662

**Published:** 2023-02-02

**Authors:** Chunzheng Wang, Jianshe Ma, Chao Cai, Ping Su

**Affiliations:** Tsinghua Shenzhen International Graduate School, Tsinghua University, Shenzhen 518055, China

**Keywords:** diffraction algorithms, sampling conditions, aliasing

## Abstract

Diffraction algorithms with adjustable magnification are dominant in holographic projection and imaging. However, the algorithms are limited by the Nyquist sampling conditions, and simulation results with inappropriate parameters sometimes appear with aliasing. At present, many diffraction algorithms have been proposed and improved, but there is a need for an overall analysis of their sampling conditions. In this paper, some classical diffraction algorithms with adjustable magnification are summarized, and their sampling conditions in the case of plane wave or spherical wave illumination are analyzed and compared, which helps to select the appropriate diffraction algorithm according to the specific parameter conditions of the simulation to avoid aliasing.

## 1. Introduction

In the applications of large field of view diffraction projection, such as advanced vehicle front-lighting [1], holographic projection [2,3], and 3D sensing [4], the generation of holograms is a key problem [5]. This process inevitably requires the simulation of the forward and backward diffraction processes of light between the initial plane and the diffraction plane; therefore, a diffraction algorithm with adjustable magnification is necessary.

**Figure 1:**
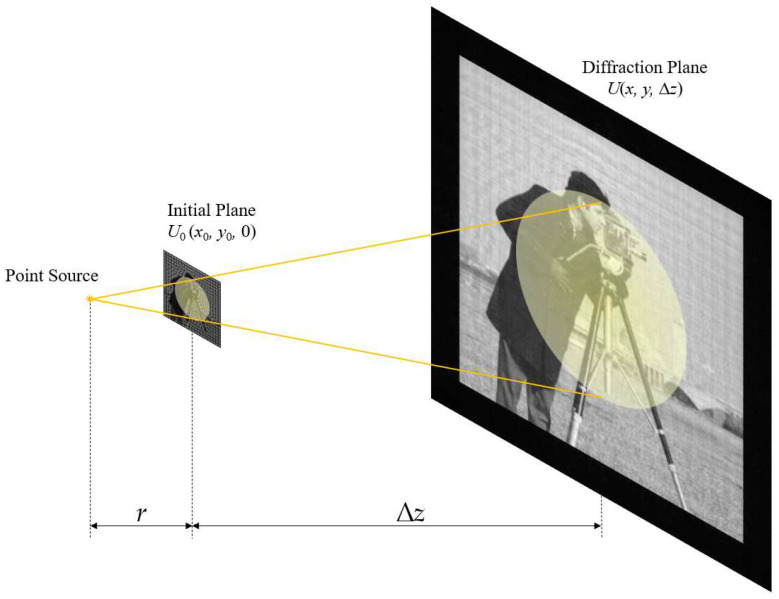
Schematic of the diffraction imaging process.

Figure 1 shows a typical diffraction process. The complex amplitude distribution of the light field on the initial plane is denoted as *U*_0_(*x*_0_, *y*_0_, 0). The complex amplitude distribution at the diffraction distance ∆*z* on the diffraction plane is denoted as *U*(*x*, *y*, ∆*z*). The diffraction process can be described by two equations [6].

(1) The angular spectrum diffraction equation:

(1) $$U\left(x,y,\Delta z\right)=F^{-1}\left\{F\left\{U_{0}\left(x_{0},y_{0},0\right)\right\}\exp\left[i\frac{2\pi }{\lambda }\Delta z\sqrt{1-{\left(\lambda f_{x_{0}}\right)}^{2}-{\left(\lambda f_{y_{0}}\right)}^{2}}\right]\right\}\;.$$

(2) When the divergence angle of the light field is small enough, Equation (1) can be converted to the following two equations under the paraxial approximation:

(2) $$U\left(x,y,\Delta z\right)=\frac{\exp\left(ik\Delta z\right)}{i\lambda \Delta z}\times \iint_{-\infty }^{+\infty }U_{0}\left(x_{0},y_{0},0\right)\exp\left\{\frac{ik}{2\Delta z}\left[{\left(x-x_{0}\right)}^{2}+{\left(y-y_{0}\right)}^{2}\right]\right\}dx_{0}dy_{0},$$

(3) $$\begin{array}{cc} U\left(x,y,\Delta z\right)= & \frac{\exp\left(ik\Delta z\right)}{i\lambda \Delta z}\exp\left[\frac{ik}{2\Delta z}\left(x^{2}+y^{2}\right)\right]\times \iint_{-\infty }^{+\infty }\left\{U_{0}\left(x_{0},y_{0},0\right)\exp\left[\frac{ik}{2\Delta z}\left(x_{0}^{2}+y_{0}^{2}\right)\right]\right\}\times \\ & \exp\left[-i2\pi \left(x_{0}\frac{x}{\lambda \Delta z}+y_{0}\frac{y}{\lambda \Delta z}\right)\right]dx_{0}dy_{0} \end{array}.$$

Since the Fourier transform can be calculated via fast Fourier transform (FFT) [6], Equation (3) is widely used in the study of diffraction problems. The single fast Fourier transform (S-FFT) [7] is a classical method for calculation of the diffraction process using FFT. This method uses FFT once to calculate the diffraction process. If we want to obtain diffraction results satisfying the sampling theorem, the number of sampling points, *N*, the initial plane size, *L*_0_, the diffraction plane size, *L*, the sampling interval, ∆*x*_0_, and the scaling factor, *m*, of *L* relative to *L*_0_ should satisfy the following condition:

(4) $$L=\frac{\lambda \Delta zN}{L_{0}}\Leftrightarrow m\Delta x_{0}^{2}N=\lambda \Delta z\;.$$

When the diffraction distance is small, the target plane size *L* will be close to 0. In order to ensure that *L* is not too small under the premise that *L*_0_ is fixed, a large number of sampling points, *N*, is necessary, which will increase the amount of calculations. However, in a large field of view diffraction projection system, the initial plane size is often much smaller than the diffraction plane size, and the use of S-FFT has great limitations. Therefore, some more flexible numerical algorithms have been developed to break the limit and make flexible calculations with adjustable magnification between the sizes of the initial plane and the diffraction plane.

One of them is the double Fresnel transform (DBFT) algorithm, which was proposed by Fucai Zhang et al. for the reconstruction of recorded digital holograms [8]. Later, an improved algorithm with similar principles was also proposed [9]. The algorithm involves two reconstruction steps implemented by conventional S-FFT, such that the magnification between the sizes of the initial plane and the diffraction plane can be adjusted flexibly by adjusting the diffraction distances of the two steps.

In the book *Numerical Simulation of Optical Wave Propagation*, the author Jason D. Schmidt mentioned an algorithm containing a scaling factor [10] (scaled angular spectrum method, SASM). Furthermore, Richard P. Muffletto et al. proposed the shifted Fresnel diffraction algorithm [11] (SFD), which can compute the Fresnel propagation of light between parallel planes with different sampling intervals. The common feature of the two algorithms is that the magnification between the sampling intervals of the initial plane and the diffraction plane can be set independently before simulation.

Although the first three diffraction algorithms are improved compared with the ordinary S-FFT in terms of sampling conditions, they are still limited. If all algorithms are not applicable to the set simulation parameters, the algorithms [12,13,14] with relatively smaller sampling limit can also be used at the expense of computing efficiency. Their mathematical principle is similar to nonuniform fast Fourier transform (NUFFT) in signal processing theory [15]. One of them is the matrix product angular spectrum method (MPASM). This algorithm was proposed by Wanli Zhao et al. in 2020 [14]. In this method, a DFT calculation based on the matrix product is realized.

The ideas of these four algorithms to break the sampling limit of S-FFT are classical and representative. Therefore, their specific sampling conditions are further analyzed in this paper. Our conclusion can also be referred to when analyzing other algorithms with similar ideas.

In simulations of the diffraction process of the light field, the Nyquist sampling condition needs to be considered. That is, the condition that the sampling frequency is not less than twice of the maximum spatial frequency in the light field distribution needs to be satisfied, otherwise aliasing will occur.

Generally, there are two kinds of aliasing results. One is that the image is divided into several parts. The other is the appearance of horizontal and vertical high brightness stripes. They darken the whole image due to brightness normalization, which is also one of the bases for judging whether the image is aliased. These two different aliasing phenomena are caused by the failure of different steps in the calculation to meet the sampling conditions.

In most diffraction algorithms, the maximum spatial frequency of the light field generally depends on the phase factors in the diffraction equation, especially the quadratic phase factors. The spatial frequency of the amplitude is generally much smaller than that of the phase, which is often not considered [6]. In the general quadratic phase factors, the spatial frequency becomes larger upon approaching the field edge. Therefore, when analyzing the sampling frequency, the spatial frequency of the phase distribution at the four corners of the rectangular field is generally taken as the maximum spatial frequency for analysis, so as to ensure that every 2π change in the phase will pass through at least two sampling points. Different diffraction algorithms involve different phase factors; therefore, their sampling conditions are also different. There is an urgent need for overall analysis of their sampling conditions.

Compared with the S-FFT algorithm with a simple calculation process, the calculation processes of these algorithms are more complex. Therefore, each step of the calculation process needs to be analyzed when analyzing the sampling conditions, and the mutual influence between the sampling limits of different parts should also be considered. In addition, compared with simple plane wave illumination, spherical wave illumination with an additional quadratic phase factor also makes the analysis of sampling conditions more complex.

Four classical diffraction algorithms are summarized in this paper: the double Fresnel transform [8] (DBFT), the scaled angular spectrum method [10] (SASM), the shifted Fresnel diffraction [11] (SFD), and the matrix product angular spectrum method [14] (MPASM). Their sampling conditions are analyzed and compared, which is helpful when selecting the appropriate diffraction algorithm according to specific simulation conditions to avoid aliasing. As a result, when using them, people often encounter aliasing due to inappropriate parameters, but do not know where and how to adjust them. Next, this paper briefly introduces the principles and analyzes the sampling conditions for each algorithm, and finally summarizes and compares them to provide important references.

## 2. Methods

Generally, in the diffraction equation, if the phase factors in the Fourier transform term do not satisfy the sampling conditions, the amplitude distribution of the simulated diffraction result will be aliased, and if the phase factors outside the Fourier transform term do not satisfy the sampling conditions, the phase distribution of the simulated diffraction result will be aliased. In display applications, while only the amplitude distribution of the diffracted result is considered, the phase factors outside the Fourier transform term are often ignored. Yet, for a specific diffraction algorithm, it is also necessary to specifically analyze which phase factors are related to the amplitude and which are related to the phase in the target plane before making a choice.

When the initial plane is illuminated by a spherical wave, a quadratic phase factor is introduced into it. The phase factor is related to the distance from the point light source to the initial plane. Generally, the phase factor is similar to the phase factors in the diffraction equation; thus, it can be combined with them to form new phase factors. Then, it is necessary to analyze the sampling conditions of the combined new phase factor. The results obtained by analyzing these phase factors separately and then taking their intersection are inaccurate.

On the basis of the discussions above, we briefly introduction the four algorithms and discuss their sampling conditions separately.

### 2.1. SFD

The SFD algorithm considers the condition that the center of the initial plane and the center of the diffraction plane are not coaxial and the sampling intervals of the two sampling planes are different. Taking the center point as a relative reference, each sampling point can be written as:

(5) $$\begin{array}{c} x_{0}=x_{0c}+\left(\frac{1}{2}+p_{0}\right)\Delta x_{0},y_{0}=y_{0c}+\left(\frac{1}{2}+q_{0}\right)\Delta y_{0}, \end{array}$$

(6) $$\begin{array}{c} x=x_{c}+\left(\frac{1}{2}+p\right)\Delta x,y=y_{c}+\left(\frac{1}{2}+q\right)\Delta y, \end{array}$$

where *p*_0_, *q*_0_, *p*, and *q* are within [−*N*/2, *N*/2 − 1] and *N* is the number of horizontal (vertical) sampling points. Only the case where the two sampling planes are square is considered here, and the sampling points of the two planes are the same. Then, according to Equation (3), we obtain:

(7) $$\begin{array}{c} U\left(p,q\right)=C\sum_{p_{0}}\sum_{q_{0}}a\left(p_{0},q_{0}\right)b\left(p-p_{0},q-q_{0}\right)\;, \end{array}$$

where

(8) $$\left\{\begin{array}{c} \begin{array}{cc} C= & \frac{\exp(ik\Delta z)}{i\lambda \Delta z}\exp\left[i\frac{k}{2\Delta z}\left(x^{2}+y^{2}\right)\right]\exp\left[-i\frac{2\pi }{\lambda \Delta z}\left(px_{0c}\Delta x+qy_{0c}\Delta y\right)\right]\\ & \exp\left[-i\frac{2\pi }{\lambda \Delta z}\left(\frac{1}{2}p\Delta x_{0}\Delta x+\frac{1}{2}q\Delta y_{0}\Delta y\right)\right]\exp\left[-i\frac{\pi }{\lambda \Delta z}\left(p^{2}\Delta x_{0}\Delta x+q^{2}\Delta y_{0}\Delta y\right)\right] \end{array}\\ \begin{array}{cc} a(p_{0},q_{0})= & U_{0}(p_{0},q_{0})\exp\left[i\frac{k}{2\Delta z}\left(x_{0}^{2}+y_{0}^{2}\right)\right]\exp\left[-i\frac{2\pi }{\lambda \Delta z}\left(x_{0}x_{c}+y_{0}y_{c}\right)\right]\\ & \exp\left[-i\frac{2\pi }{\lambda \Delta z}\left(\frac{1}{2}p_{0}\Delta x_{0}\Delta x+\frac{1}{2}q_{0}\Delta y_{0}\Delta y\right)\right]\exp\left[-i\frac{\pi }{\lambda \Delta z}\left(p_{0}^{2}\Delta x_{0}\Delta x+q_{0}^{2}\Delta y_{0}\Delta y\right)\right]\\ b(p_{0},q_{0})= & \exp\left[i\frac{\pi }{\lambda \Delta z}\left(p_{0}^{2}\Delta x_{0}\Delta x+q_{0}^{2}\Delta y_{0}\Delta y\right)\right] \end{array} \end{array}\right..$$

Equation (7) contains a convolution operation and can be converted into a Fourier transform operation:

(9) $$U\left(p,q\right)=C\cdot F^{-1}\left\{F\left[a\left(p_{0},q_{0}\right)\right]F\left[b\left(p_{0},q_{0}\right)\right]\right\}\;.$$

Then, FFT can be used, and the calculation speed will be greatly accelerated. A total of three FFT operations are required. This algorithm has been used in holographic projections without lenses [16] and 3D holography [17].

In addition, Tomoyoshi Shimobaba’s group further proposed an improved algorithm ARSS (aliasing reduced Fresnel diffraction with scale and shift operations) to address the drawback of SFD that aliasing will occur with a short diffraction distance [18]. By introducing a rectangular window, the algorithm eliminates the aliasing phenomenon with a short diffraction distance. They used the ARSS in a virtual converged spherical wave computer-generated holographic system [19], in which the method of cyclic iteration was used to improve the clarity of the reconstructed hologram. However, in order to facilitate comparison with other algorithms, only sampling conditions of the basic algorithm are analyzed.

SFD determines the scaling factor before deriving the diffraction process, and then extracts the convolution operation, so that FFT can be used for fast calculation.

The quadratic phase factors of *a*(*p*_0_, *q*_0_) and *b*(*p*_0_, *q*_0_) in Equation (9) are mainly analyzed. Here, in order to keep the simulation conditions consistent with other algorithms, only the case where the centers of the initial plane and diffraction plane are coaxial is considered. The scaling factor is still assumed to be *m*. Next, as shown in Figure 1, we consider the phase factor introduced by the spherical wave for illumination, which will also affect the sampling conditions.

The phase factor introduced by the illumination spherical wave *P_div_* is expressed as:

(10) $$\begin{array}{c} P_{div}=\exp\left(i\phi_{div}\right)=\exp\left(ik\frac{x_{0}^{2}\;+\;y_{0}^{2}}{2r}\right). \end{array}$$

It should be noted that the spherical wave here is not necessarily a divergent spherical wave illuminated by a point light source. This is only true when *r* is > 0. When *r* is < 0, the spherical wave is convergent. When *r* tends to +∞, the incident light tends to the plane wave. Here, we only analyze the case where *r* is > 0 or *r* tends to +∞.

In order to satisfy the sampling theorem, the sampling frequency should be higher than twice the maximum spatial frequency. Generally, the phase frequency is much higher than the amplitude frequency. Therefore, only the phase factor in the diffraction equation is considered. Given that the maximum spatial frequency is located at the edge, where the sampling frequency shall satisfy the sampling theorem, for *a*(*p*_0_, *q*_0_), we have:

(11) $$\Delta x_{0}\leq \left|\frac{\Delta zr\lambda }{L_{0}(r-mr+\Delta z)}\right|\;.$$

The analysis of the quadratic phase factor in *b*(*p*_0_, *q*_0_) shows that:

(12) $$\Delta x_{0}\leq \frac{\Delta z\lambda }{mL_{0}}\;.$$

**Figure 2:**
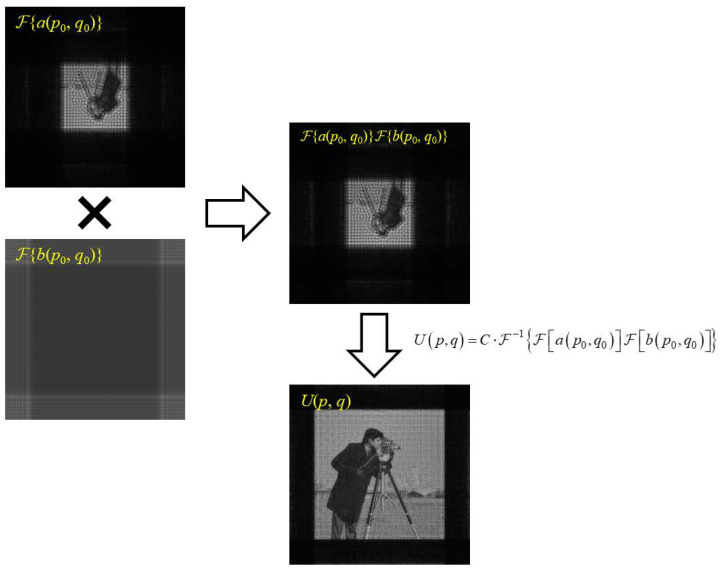
Schematic of sampling condition analysis process of SFD with amplitude distributions of some factors involved.

Next, further analysis is conducted to observe the results of ℱ{*a*(*p*_0_, *q*_0_)}, ℱ{*b*(*p*_0_, *q*_0_)}, and ℱ{*a*(*p*_0_, *q*_0_)}ℱ{*b*(*p*_0_, *q*_0_)}. Here, taking ∆*z* = 500 mm as an example, the analysis process is shown in Figure 2.

In Figure 2, the aliasing phenomenon occurs at the edge of ℱ{*b*(*p*_0_, *q*_0_)}, but the final diffraction result is normal, because there is an invalid blank area around ℱ{*a*(*p*_0_, *q*_0_)}. In addition, in the process of multiplying ℱ{*b*(*p*_0_, *q*_0_)}, the aliased part in ℱ{*b*(*p*_0_, *q*_0_)} does not affect the effective area at the center of ℱ{*a*(*p*_0_, *q*_0_)}; hence, it does not affect the final result. According to this discussion, the analysis of the quadratic phase factor in *b*(*p*_0_, *q*_0_) can be adjusted. It is not necessary to satisfy the sampling conditions at the farthest edge of the field, as long as the sampling conditions are satisfied at the edge of the effective area of ℱ{*a*(*p*_0_, *q*_0_)}.

Therefore, we need to calculate the range of the effective area in ℱ{*a*(*p*_0_, *q*_0_)}. From the form of *a*(*p*_0_, *q*_0_), we can get that ℱ{*a*(*p*_0_, *q*_0_)} is actually the propagation result of the initial plane illuminated by a spherical wave of radius *r* through the distance ∆*z*/(1 − *m*), which will scale the size of image by [*r*(*m* − 1) − ∆*z*]/[*r*(*m* − 1)] times. In addition, after a single FFT calculation, the sampling range is scaled by [*N*∆*x*_0_^2^(*m* − 1)]/(*λ*∆*z*) times. Given these two scales, the scale of the range of the effective area relative to the original image should be *N*∆*x*_0_^2^[*r*(*m* − −1) − ∆*z*]/(*λr*∆*z*), according to which we can scale N in the sampling conditions obtained from the analysis of the quadratic phase factor in *b*(*p*_0_, *q*_0_) to obtain a new sampling condition. It should be noted here that, in order to ensure that the amplitude of ℱ{*b*(*p*_0_, *q*_0_)} does not appear aliasing at the boundary of the effective area, *b*(*p*_0_, *q*_0_) is required to satisfy the sampling conditions in the diagonal direction at the corners of the effective area, and its sampling interval is $\sqrt{2}$ times of the horizontal or vertical interval. Then, we obtain:

(13) $$\Delta x_{0}\leq \frac{\Delta z\lambda }{L_{0}}\sqrt{\frac{r}{\sqrt{2}m(mr-r-\Delta z)}}\;.$$

Next, we analyze the phase factor in ℱ{*a*(*p*_0_, *q*_0_)}ℱ{*b*(*p*_0_, *q*_0_)}. *U*(*p*_0_, *q*_0_) and the linear phase factor in *a*(*p*_0_, *q*_0_) have little influence on ℱ{*a*(*p*_0_, *q*_0_)}; thus, they can be ignored. Then, the phase factor of *a*(*p*_0_, *q*_0_) can be regarded as exp{*i*π[(1 − *m*)*r* + ∆*z*](*x*_0_^2^ + *y*_0_^2^)/(*λr*∆*z*)}, and the phase factor of ℱ{*a*(*p*_0_, *q*_0_)} can be calculated as exp{*i*π(*λr*∆*z*)(*f_x_*^2^ + *f_y_*^2^)/[(*m* − 1)*r* − ∆*z*]}. Meanwhile, the phase factor in ℱ{*b*(*p*_0_, *q*_0_)} can be calculated as exp{−*i*π*λ*∆*z*(*f_x_*^2^ + *f_y_*^2^)/*m*}. Multiplying the two phase factors, we obtain exp{*i*π*λ*∆*z*(*r*/[(*m* − 1)*r* − ∆*z*] − 1/*m*)(*f_x_*^2^ + *f_y_*^2^)}, which meets the sampling conditions at the boundary of the effective area. Then, we obtain:

(14) Δz≤(m−1)r .

Since Equation (13) is more restrictive than Equation (11), the final adjusted sampling conditions are:

(15) $$\Delta x_{0}\leq \frac{\Delta z\lambda }{L_{0}}\sqrt{\frac{r}{\sqrt{2}m(mr-r-\Delta z)}}\;,\;\Delta z\leq (m-1)r\;.$$

Next, we performed simulations to verify this sampling condition for the SFD algorithm. Two verifications were performed. One is to adjust the propagation distance ∆*z* with other parameters fixed, and the other is to adjust ∆*x*_0_ and *N* with other parameters and initial plane size (*L*_0_ = *N*∆*x*_0_) fixed. For the first verification, the simulation parameters are set as shown in Table 1. According to Equation (15) and the simulation parameters, the sampling condition is satisfied when 450 mm ≤ ∆*z* ≤ 750 mm.

**Figure 3:**
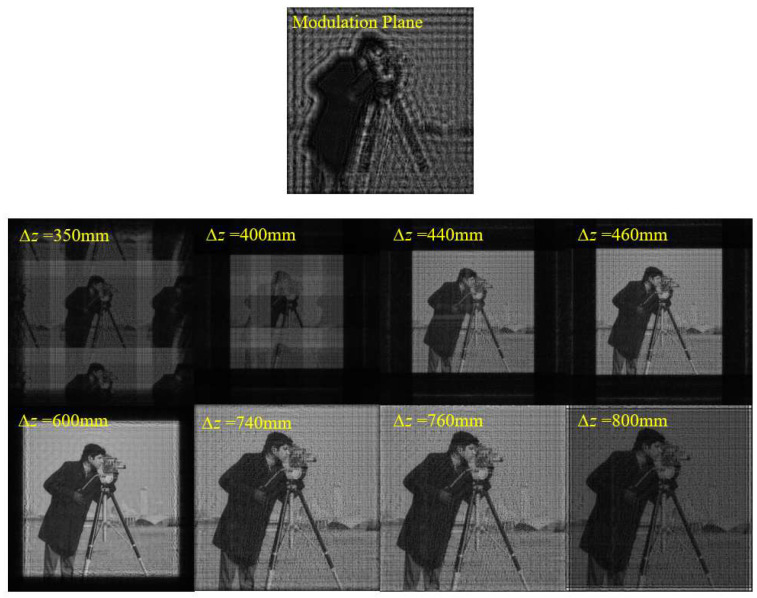
Initial (modulation) plane and calculated amplitude distributions on the diffraction plane with different ∆*z* using SFD.

Figure 3 shows the amplitude distributions on the diffraction plane with different ∆*z*. It can be seen that the calculated sampling range is consistent with the simulation results. There is a hologram on the initial plane. In theory, with the condition of *r* = 150 mm given in Table 1, a clear image in focus will be obtained on the diffraction plane 600 mm behind it. All verifications in this paper use the hologram as the initial plane. When ∆*z* decreases to 450 mm, horizontal and vertical bright stripes begin to appear in the middle of the image, and the image brightness starts to decrease. With a further decrease in ∆*z*, the phenomenon of aliasing becomes more serious. Multiple images overlap and interlace, and the image brightness lowers. When ∆*z* increases to 750 mm, bright stripes parallel to the edges begin to appear around the image, and the image brightness starts to decrease. With the further increase in ∆*z*, the aliasing becomes more serious, and the image brightness lowers.

For the second verification, the simulation parameters are set as shown in Table 2. According to Equation (15) and the simulation parameters, the sampling condition is satisfied when ∆*x*_0_ ≤ 15.086 μm.

**Figure 4:**
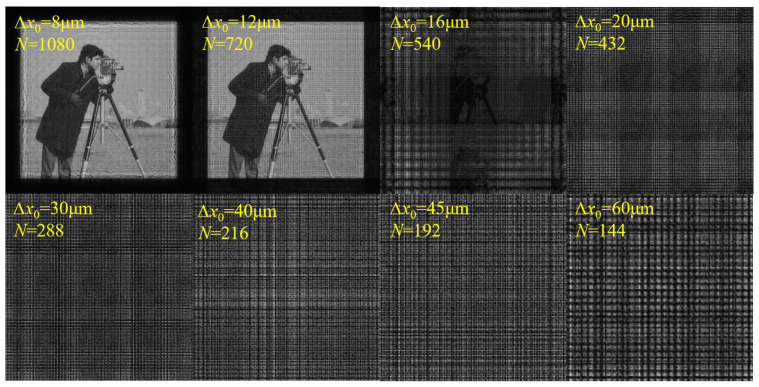
Calculated amplitude distributions on the diffraction plane with different ∆*x*_0_ and *N* using SFD.

Figure 4 shows the amplitude distributions on the diffraction plane with different ∆*x*_0_ and *N*. The calculated sampling range is also consistent with the simulation results. When ∆*x*_0_ increases to 15.086 μm, multiple images overlap and interlace, horizontal and vertical bright stripes begin to appear, and the image brightness starts to decrease. With the further increase in ∆*x*_0_, the phenomenon of aliasing becomes more serious, and the image becomes illegible. The results prove that the sampling conditions are correct from another perspective. In addition, this sampling condition is also applicable to the Fresnel–Bluestein algorithm, the mathematical essence of which is similar to that of SFD [20].

### 2.2. SASM

In the book *Numerical Simulation of Optical Wave Propagation* [10], the author Jason D. Schmidt mentioned an algorithm, which assumes that both sides are coaxial, and also introduces the scaling factor *m*, but uses FFT once less than SFD. The author uses angular spectrum propagation to define this method because its calculation form is similar to the angular spectrum form of the Fresnel diffraction integral. This method still uses a paraxial approximation, so it is not strictly equivalent to the true angular spectrum form.

In order to simplify the derivation process of SASM, the following operators are introduced:

(16) $$\left\{\begin{array}{l} Q\left[c,r\right]\left\{U\left(r\right)\right\}\equiv \exp(i\frac{k}{2}c|r{|}^{2})U\left(r\right)\\ F\left[r,f\right]\left\{U\left(r\right)\right\}\equiv \int_{-\infty }^{+\infty }U\left(r\right)\exp(-i2\pi f\cdot r)dr\;,\\ F^{-1}\left[f,r\right]\left\{U\left(f\right)\right\}\equiv \int_{-\infty }^{+\infty }U\left(f\right)\exp(i2\pi f\cdot r)df \end{array}\right.$$

where the operators *Q*[*c*, **r**], $F\left[r,\;f\right]$, and $F^{-1}\left[r,\;f\right]$ in the equation indicate multiplication by the phase factor, Fourier transform, and inverse Fourier transform, respectively. The scalar forms of the vector symbol are **r** = (*x*, *y*) and **f** = (*f_x_*, *f_y_*), representing the spatial coordinate and the frequency domain coordinate, respectively. Next is the derivation of SASM. First, the Fresnel diffraction equation can be written as:

(17) $$U\left(r\right)=\frac{1}{i\lambda \Delta z}\int_{-\infty }^{+\infty }U_{0}\left(r_{0}\right)\exp(i\frac{k}{2\Delta z}{\left|r-r_{0}\right|}^{2})dr_{0}\;,$$

where ∆*z* is the diffraction distance and **r**_0_ and **r** denote the coordinate vectors at the initial plane and the diffraction plane, respectively. Then, a size scaling factor *m* of the diffraction plane relative to the initial plane is introduced, and the identity transformation can be performed:

(18) $$\begin{array}{ll} {\left|r-r_{0}\right|}^{2} & =r^{2}-2r\cdot r_{0}+r_{0}^{2}\\ & =\left(r^{2}+\frac{r^{2}}{m}-\frac{r^{2}}{m}\right)-2r\cdot r_{0}+\left(r_{0}^{2}+mr_{0}^{2}-mr_{0}^{2}\right)\\ & =m\left[{\left(\frac{r}{m}\right)}^{2}-2\left(\frac{r}{m}\right)\cdot r_{0}+r_{0}^{2}\right]+\left(1-\frac{1}{m}\right)r^{2}+(1-m)r_{0}^{2}\\ & =m{\left|\frac{r}{m}-r_{0}\right|}^{2}-\left(\frac{1-m}{m}\right)r^{2}+(1\;-\;m)r_{0}^{2} \end{array}.$$

With Equation (17), we can further obtain:

(19) $$U\left(r\right)=\frac{\exp(-i\frac{k}{2\Delta z}\left(\frac{1\;-\;m}{m}\right)r^{2})}{i\lambda \Delta z}\int_{-\infty }^{+\infty }U_{0}\left(r_{0}\right)\exp(i\frac{k}{2\Delta z}\left(1-m\right)r_{0}^{2})\exp(i\frac{km}{2\Delta z}{\left|\frac{r}{m}-r_{0}\right|}^{2})dr_{0}\;.$$

Finally, the convolution theorem can be used to obtain *U*(**r**_2_) as:

(20) $$\begin{array}{cc} U\left(r\right)= & Q\left[\frac{m\;-\;1}{m\Delta z},r\right]F^{-1}\left[f_{0},\frac{r}{m}\right]Q\left[-\frac{\lambda^{2}\Delta z}{m},f_{0}\right]\times \\ & F\left[r_{0},f_{0}\right]Q\left[\frac{1\;-\;m}{\Delta z},r_{0}\right]\frac{1}{m}\left\{U_{0}\left(r_{0}\right)\right\} \end{array}.$$

In this algorithm, the scaling factor is determined before the diffraction process is derived, and the convolution operation is extracted such that FFT can be used for fast calculations.

This is similar to SFD in essence. The difference is that, for the Fourier transform of *b* in SFD and *h* in SASM, SFD calculates the Fourier transform, while SASM directly uses the phase factor of the calculation result. The use of FFT in the calculation process is an important factor influencing the sampling conditions; therefore, there are still differences between them. That is, SASM does not have to satisfy the second sampling condition in Equation (13) of SFD, which is obtained from the analysis of *b*(*p*_0_, *q*_0_).

The final sampling conditions are as follows:

(21) $$\Delta x_{0}\leq \frac{\Delta zr\lambda }{L_{0}(mr-r-\Delta z)}\;,\;\Delta z\leq (m-1)r\;.$$

**Figure 5:**
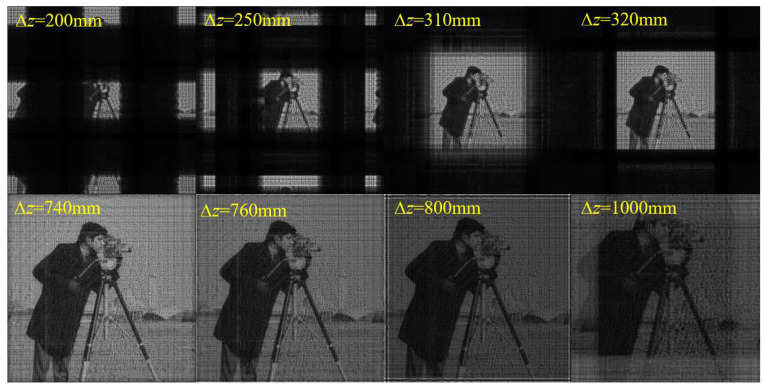
Calculated amplitude distributions on the diffraction plane with different ∆*z* using SASM.

Next, we performed simulations to verify these sampling conditions for the SASM algorithm. The two verifications were similar to those of SFD. For the first, the simulation parameters were also set as shown in Table 1. According to Equation (21) and the simulation parameters, the sampling condition is satisfied when 316 mm ≤ ∆*z* ≤ 750 mm. Figure 5 shows the amplitude distributions of the diffraction plane with different ∆*z*. It can be seen that the calculated sampling range is consistent with the simulation results. Within the calculated range, the image is only scaled. Beyond this range, aliasing occurs. When ∆*z* is large, the result of SASM is the same as that of SFD. When ∆*z* is reduced to 316 mm, the edge parts of the image start to separate from the center part, and the image is divided into nine parts, which is different from the common effect of aliasing containing replications, and is caused by the violation of the Nyquist sampling theorem by the second Fourier transform in Equation (20). With a further decrease in ∆*z*, the segmented part becomes larger and farther away from the center.

For the second verification, the simulation parameters were set as shown in Table 2. According to Equation (21) and the simulation parameters, the sampling condition is satisfied when ∆*x*_0_ ≤ 43.944 μm.

**Figure 6:**
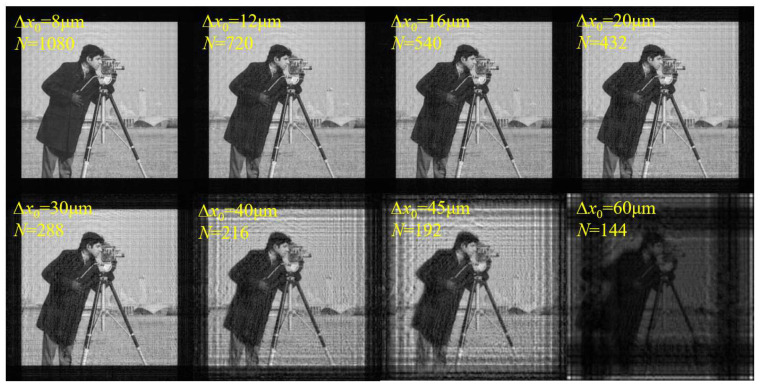
Calculated amplitude distributions on the diffraction plane with different ∆*x*_0_ and *N* using SASM.

Figure 6 shows the amplitude distributions on the diffraction plane with different ∆*x*_0_ and *N*. The calculated sampling range is also consistent with the simulation results. When ∆*x*_0_ increases to 43.944 μm, the edge of the image begins to blur, and the image brightness starts to decrease. With a further increase in ∆*x*_0_, the phenomenon of aliasing becomes more serious; the edge parts of the image separate from the center part evidently such that the image is divided into nine parts and the whole image becomes blurred. It can be seen that the sampling conditions of SASM are not as strict as those of SFD due to the lack of the sampling limit for *b*(*p*_0_, *q*_0_).

### 2.3. DBFT

**Figure 7:**
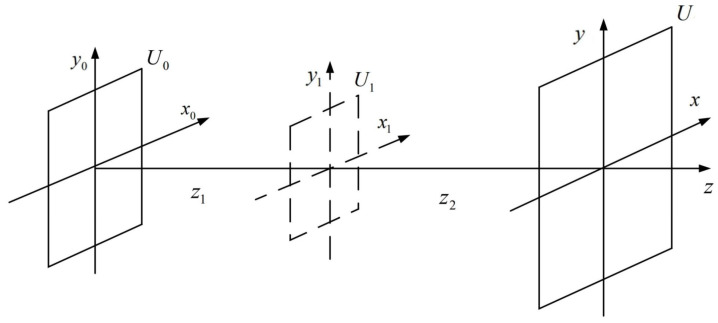
Schematic of DBFT.

Figure 7 is the schematic of DBFT. *U*_0_(*x*_0_, *y*_0_) and *U*(*x*, *y*) represent the complex amplitude distribution of light field in the initial plane and the diffraction plane, respectively, while *U*_1_(*x*_1_, *y*_1_) is the complex amplitude distribution of the light field in a virtual intermediate plane that does not physically exist, which is indicated by the dotted lines. The diffraction process of the light field from *U*_0_(*x*_0_, *y*_0_) to *U*_1_(*x*_1_, *y*_1_), and from *U*_1_(*x*_1_, *y*_1_) to *U*(*x*, *y*) is taken into account, such that the size of the diffraction plane can be adjusted by changing the position of the virtual intermediate plane.

The sampling intervals on the three planes are ∆*x*_0_ = ∆*y*_0_ = *L*_0_/*N*, ∆*x*_1_ = ∆*y*_1_ = *L*_1_/*N*, and ∆*x* = ∆*y* = *L*/*N*, where *L* represents the sampling range and *N* represents the number of sampling points. The sampling range of the frequency domain (*L*_*u*_, *L*_*v*_) has the following mathematical relation with the spatial domain sampling interval in the discrete Fourier transform (DFT):

(22) $$\begin{array}{c} L_{u}=\frac{1}{\Delta x},L_{v}=\frac{1}{\Delta y}\;. \end{array}$$

Therefore, for the three planes, the following relations can be obtained:

(23) $$\begin{array}{c} \frac{L_{1}}{\lambda z_{1}}=\frac{1}{\Delta x_{0}}=\frac{N}{L_{0}},\frac{L}{\lambda z_{2}}=\frac{1}{\Delta x_{1}}=\frac{N}{L_{1}}\;. \end{array}$$

Then, we have the following relation:

(24) $$\begin{array}{c} L_{1}=\frac{N\lambda z_{1}}{L_{0}},L=\frac{N\lambda z_{2}}{L_{1}}\;. \end{array}$$

The relation between *L*_0_ and *L* is:

(25) $$\begin{array}{c} L=\left|\frac{z_{2}}{z_{1}}\right|L_{0}\;. \end{array}$$

So far, the derivation of DBFT has been completed. The core idea to solve the sampling problem is as follows: in single FFT, the sampling interval in the spatial domain and that in the frequency domain are reciprocal; hence, the size *L* is not large enough. However, with double FFTs, a positive proportional relation will be obtained after two reciprocal relations, as shown in Equation (25), which is applicable to the diffraction process.

It is worth noting that the absolute value is used in Equation (25), which indicates that the distance parameter can take a negative value, i.e., the intermediate virtual plane does not have to be between the initial plane and the diffraction plane but can also be placed on the left side of the initial plane or the right side of the diffraction plane. For example, the double sampling Fresnel (DSF) algorithm with similar mathematical essence places the intermediate plane at the point light source of the spherical wave for illumination [21].

From *U*_0_(*x*_0_, *y*_0_) to *U*_1_(*x*_1_, *y*_1_),

(26) $$U_{1}\left(x_{1},y_{1}\right)=\exp\left[\frac{ik}{2z_{1}}\left(x_{1}^{2}+y_{1}^{2}\right)\right]F\left\{U_{0}\left(x_{0},y_{0}\right)\exp\left[\frac{ik}{2z_{1}}\left(x_{0}^{2}+y_{0}^{2}\right)\right]\right\}\;.$$

The phase factor outside the Fourier transform term only affects the phase distribution of *U*_1_(*x*_1_, *y*_1_); thus, it is not considered. Only the phase factor exp[(*ik*/2*z*_1_)(*x*_0_^2^ + *y*_0_^2^)] inside the Fourier transform term is considered. Thus, we obtain the sampling condition |*z*_1_| ≥ (∆*x*_0_^2^*N*)/*λ*.

From *U*_1_(*x*_1_, *y*_1_) to *U*(*x*, *y*),

(27) $$U\left(x,y\right)=\exp\left[\frac{ik}{2z_{2}}\left(x^{2}+y^{2}\right)\right]F\left\{U_{1}\left(x_{1},y_{1}\right)\exp\left[\frac{ik}{2z_{2}}\left(x_{1}^{2}+y_{1}^{2}\right)\right]\right\}\;.$$

Similarly, we obtain the sampling condition |*z*_2_| ≥ (∆*x*_1_^2^*N*)/*λ*.

Next, further analysis is conducted. The spherical wave phase factor can be combined with the phase factor in the diffraction equation. In addition, the phase factor of *U*_1_(*x*_1_, *y*_1_) can also be combined with the phase factor in the Fourier transform term in the second step. The phase factor of *U*_1_(*x*_1_, *y*_1_) is composed of two parts, one is the phase factor outside the Fourier transform term, and the other is the phase factor of the new term obtained by the Fourier operation of the part inside the Fourier transform term. The influence of the amplitude term *U*_0_(*x*_0_, *y*_0_) in the Fourier transform term can be ignored; hence, the phase factor of the new term obtained after calculation is exp{−*i*π*r*(*x*_1_^2^ + *y*_1_^2^)/[*λz*_1_(*r* + *z*_1_)]}.

In addition, similar to the SFD analysis process, the phase factor inside the Fourier transform term in *U*(*x*, *y*) only has to satisfy the sampling conditions at the boundary of effective area of *U*_1_(*x*_1_, *y*_1_). The calculation shows that the scale of the range of the effective area relative to the original image is *N*∆*x*_0_^2^(*r* + *z*_1_)/(*λrz*_1_). Therefore, the sampling conditions of the combined phase factor can be analyzed.

The final sampling conditions can be obtained as

(28) $$\left|\frac{rz_{1}}{r+z_{1}}\right|\geq \frac{\Delta x_{0}^{2}N}{\lambda }\;,\left|\frac{z_{1}(r+z_{1}+z_{2})}{rz_{2}}\right|\leq 1\;.$$

Next, we performed simulations to verify this sampling condition for the DBFT algorithm. The simulation parameters were set as shown in Table 1. According to Equation (28) and the simulation parameters, the sampling condition is satisfied when −150 mm ≤ *z*_1_ ≤ −63 mm for any ∆*z =* (*z*_1_ *+ z*_2_) > 0 mm.

**Figure 8:**
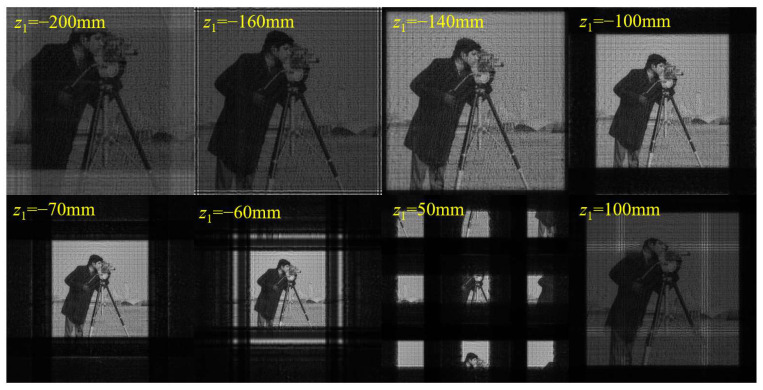
Calculated amplitude distributions on diffraction plane with different *z*_1_ and fixed ∆*z* = *z*_1_ + *z*_2_ = 750 mm using DBFT.

Figure 8 shows the amplitude distributions of the diffraction plane with different *z*_1_. It can be seen that the calculated sampling range is consistent with the simulation results. When *z*_1_ is reduced to −150 mm, bright stripes parallel to the edge begin to appear around the image, and the image brightness starts to decrease. With a further reduction in *z*_1_, the aliasing becomes more serious and the image brightness becomes lower. When *z*_1_ is increased to −63 mm, the edge part of the image starts to separate from the center part, and the image is divided into nine parts. With a further increase in *z*_1_, the segmented part becomes larger and further away from the center. When *z*_1_ exceeds 97 mm, horizontal and vertical bright stripes appear in the middle of the image and the image brightness decreases.

Next, in order to keep the same form with the sampling conditions of other algorithms for easy comparison, *z*_1_ and *z*_2_ in the sampling conditions are expressed in terms of *m* and ∆*z* according to Equation (25). Here, two cases are considered. One is *z*_2_/*z*_1_ = *m* and the other is *z*_2_/*z*_1_ = −*m*. For the former, the final sampling condition after conversion is:

(29) $$\Delta x_{0}\leq \frac{\Delta zr\lambda }{L_{0}(r+mr+\Delta z)}\;\Delta z\leq \left(m-1\right)r\;.$$

For the latter, the final sampling condition after conversion is:

(30) $$\Delta x_{0}\leq \frac{\Delta zr\lambda }{L_{0}(mr-r-\Delta z)}\;\Delta z\leq \left(m-1\right)r\;.$$

It can be seen that the sampling condition of the latter is better, which is consistent with that of SASM. Therefore, in order to avoid aliasing as much as possible, the virtual intermediate plane should be placed on the left side of the initial plane rather than between the initial plane and the diffraction plane when the algorithm is used for large field of view diffraction calculations. Next, two verifications similar to those of other algorithms were performed. Here, *m* is fixed, which means that the ratio of *z*_2_/*z*_1_ is fixed. In addition, *z*_1_ is always negative while *z*_2_ is always positive.

For the first verification, the simulation parameters were also set as shown in Table 1. According to Equation (30) and the simulation parameters, the sampling condition is satisfied when 316 mm ≤ ∆*z* ≤ 750 mm.

For the second verification, the simulation parameters were set as shown in Table 2. According to Equation (30) and the simulation parameters, the sampling condition is satisfied when ∆*x*_0_ ≤ 43.944 μm.

**Figure 9:**
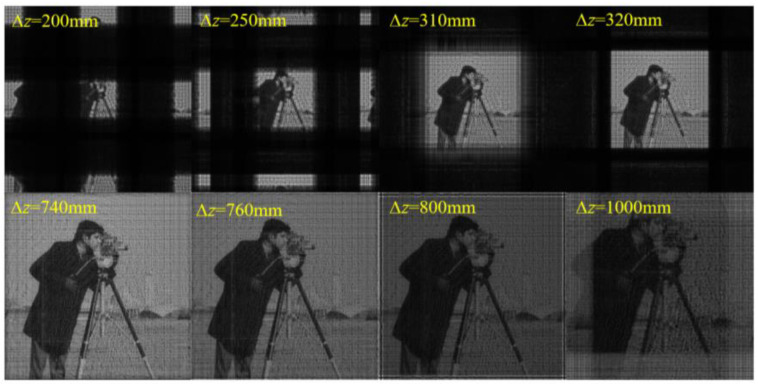
Calculated amplitude distributions on the diffraction plane with different ∆*z* using DBFT.

**Figure 10:**
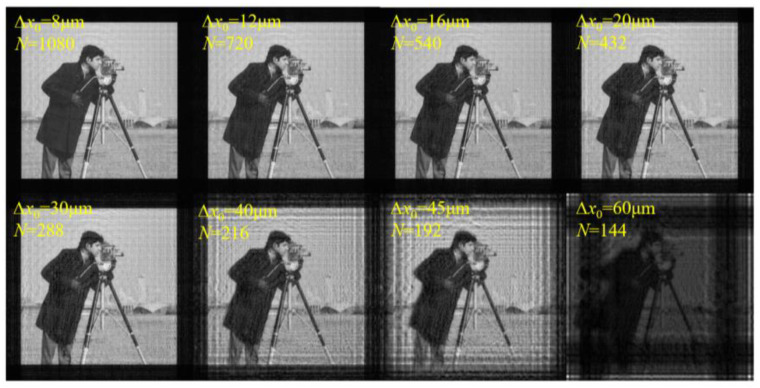
Calculated amplitude distributions on the diffraction plane with different ∆*x*_0_ and *N* using DBFT.

It can be seen from Figure 9 and Figure 10 that not only are the final sampling conditions of DBFT and SASM consistent, but their verification results with the same parameters are also consistent, which shows that although the overall ideas of the two algorithms are completely different, their mathematical essence is similar.

### 2.4. MPASM

It can be seen that there are still sampling restrictions for the above three algorithms. If all algorithms are not applicable, the matrix product angular spectrum method (MPASM) can be considered. This algorithm was proposed by Wanli Zhao et al. in 2020 [14]. In this method, the calculation of the true angular-spectrum form without paraxial approximation based on matrix product is realized.

To get rid of the limitation ∆*f_x_* = 1/(*N*∆*x*), this algorithm uses the matrix product instead of FFT to calculate the diffraction process. Moreover, the frequency domain range can be adjusted by adjusting the number of frequency domain sampling points to satisfy the sampling conditions. Therefore, there is no sampling limit in theory, but the cost is that the calculation time is increased; furthermore, if the number of points is increased to satisfy the sampling conditions, the calculation time will be further increased.

The diffraction process is calculated using the angular spectrum formula:

(31) $$U\left(x,y\right)=F^{-1}\left\{F\left\{U_{0}\left(x_{0},y_{0}\right)\right\}\cdot H\left(f_{x_{0}},f_{y_{0}}\right)\right\}\;,$$

where

(32) $$\begin{array}{c} H\left(f_{x_{0}},f_{y_{0}}\right)=\exp\left[i\frac{2\pi }{\lambda }\Delta z\sqrt{1-{\left(\lambda f_{x_{0}}\right)}^{2}-{\left(\lambda f_{y_{0}}\right)}^{2}}\right]\;. \end{array}$$

After replacing FFT with matrix product operation, the diffraction equation can be converted into:

(33) $$U\left(x,y\right)=\frac{1}{MNK_{f_{x_{0}}}K_{f_{y_{0}}}S_{1}S_{2}}M_{y,f_{y_{0}}}\times \left\{\left[M_{y_{0},f_{y_{0}}}^{T}U_{0}\left(x_{0},y_{0}\right)M_{f_{x_{0}},x_{0}}^{T}\right]\cdot H\left(f_{x_{0}},f_{y_{0}}\right)\right\}M_{f_{x_{0}},x}\;,$$

where

(34) $$\begin{array}{lll} M_{f_{x_{0}},x_{0}} & = & \exp\left(-i2\pi f_{x_{0}}{}^{T}x_{0}\right)\\ & = & \Delta x_{0}\frac{2f_{x_{0}{}_{MAX}}}{K_{f_{x_{0}}}n}\times \\ & & \left[\begin{array}{cccc} \exp\left[-i2\pi \left(-\frac{n}{2}\right)\left(-\frac{n}{2}\right)\right] & \exp\left[-i2\pi \left(-\frac{n}{2}\right)\left(-\frac{n}{2}+1\right)\right] & \cdots & \exp\left[-i2\pi \left(-\frac{n}{2}\right)\left(\frac{n}{2}-1\right)\right]\\ \exp\left[-i2\pi \left(-\frac{n}{2}+1\right)\left(-\frac{n}{2}\right)\right] & \exp\left[-i2\pi \left(-\frac{n}{2}+1\right)\left(-\frac{n}{2}+1\right)\right] & \cdots & \exp\left[-i2\pi \left(-\frac{n}{2}+1\right)\left(\frac{n}{2}-1\right)\right]\\ \vdots & \vdots & \ddots & \vdots \\ \exp\left[-i2\pi \left(\frac{n}{2}-1\right)\left(-\frac{n}{2}\right)\right] & \exp\left[-i2\pi \left(\frac{n}{2}-1\right)\left(-\frac{n}{2}+1\right)\right] & \cdots & \exp\left[-i2\pi \left(\frac{n}{2}-1\right)\left(\frac{n}{2}-1\right)\right] \end{array}\right] \end{array}$$

where *K_fx_*_0_ is the scaling factor of the frequency domain plane sampling interval and *S*_1_ and *S*_2_ are the scaling factors of the sampling points in the frequency domain.

So far, the formula of MPASM has been obtained. This formula can be directly applied using MATLAB. Since FFT is no longer used, the sampling will not be limited by it. The disadvantages are also obvious; the computational complexity of FFT is O(*N*^2^log(*N*^2^)), while that of MPASM is O(2*N*^2.4^) [14]. When the number of sampling points is more than 300, the computational complexity of MPASM will exceed that of FFT.

For the phase factor within the transfer function:

(35) $$\begin{array}{c} \varphi =\frac{2\pi }{\lambda }\Delta z\sqrt{1-{\left(\lambda f_{x_{0}}\right)}^{2}}\;, \end{array}$$

conditions for satisfying the sampling theorem are as follows:

(36) $$\begin{array}{c} \frac{1}{2\pi }\left|\frac{\partial \varphi }{\partial f_{x_{0}}}\right|\leq \frac{1}{2\Delta f_{x_{0}}}\;. \end{array}$$

**Figure 11:**
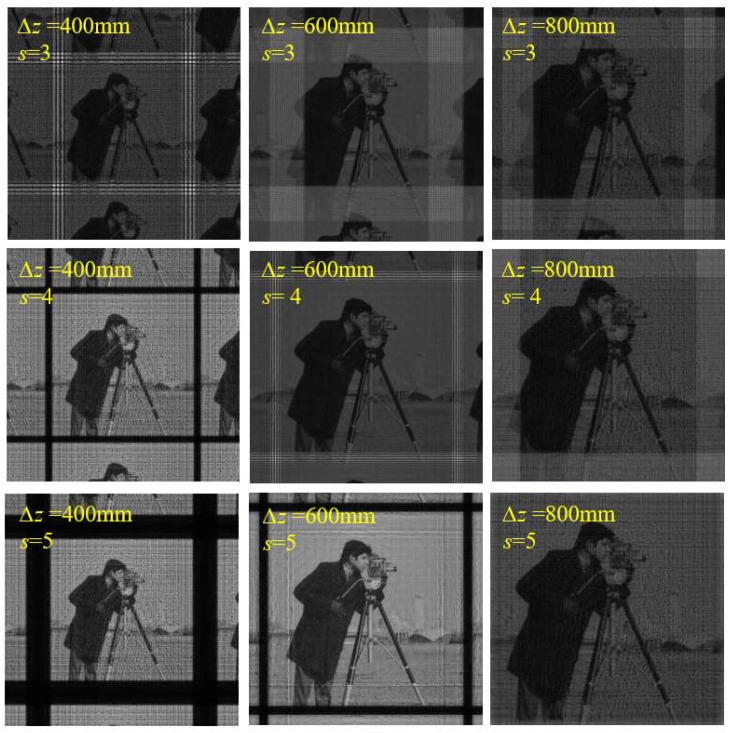
Calculated amplitude distributions on diffraction plane with different ∆*z* and different *s* using MPASM.

**Figure 12:**
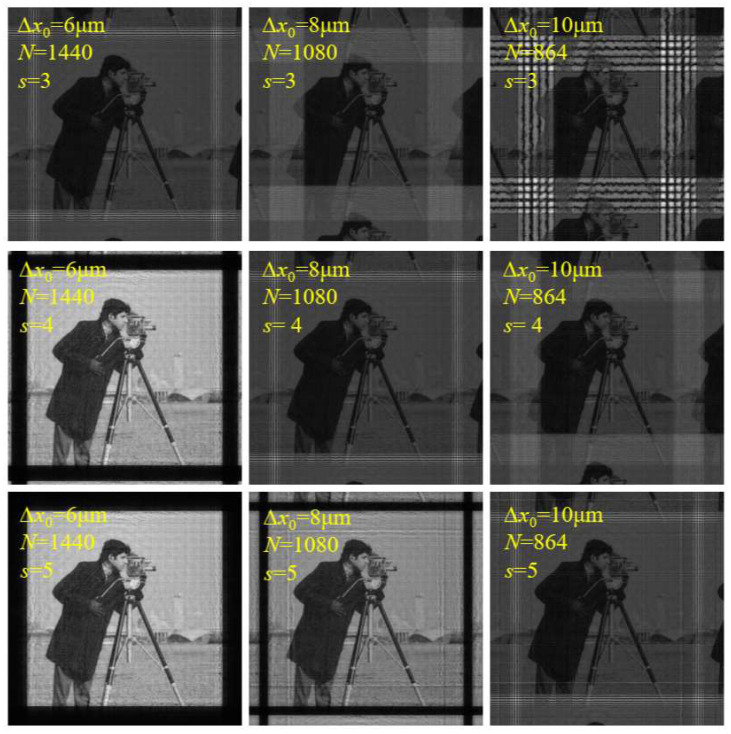
Calculated amplitude distributions on diffraction plane with different ∆*x*_0_ and different *s* using MPASM.

The maximum frequency is calculated as:

(37) $$\begin{array}{c} f_{MPASM_{-}MAX}=\frac{\sqrt{\sqrt{\left(sN\lambda^{4}\right)+64{\left(sN\lambda \Delta z\right)}^{2}}-{\left(sN\lambda \right)}^{2}}}{4\sqrt{2}\Delta z\lambda }\;, \end{array}$$

where *S*_1_ = *S*_2_ = *s*, which controls the number of sampling points. When *s* = 1, Equation (37) expresses the effective sampling range of FFT, and the sampling range decreases with the increase in the diffraction distance. When *s* > 1, it means that the number of sampling points is increased, and it can be applied to cases where the diffraction distance is relatively large. However, at the same time, it will lead to an increase in computational complexity. The simulation results are shown in Figure 11 and Figure 12. It can be seen that when *s* is small, the sampling conditions of the algorithm are relatively strict, and aliasing is easy to occur. With an increase in *s*, the sampling condition of the algorithm becomes looser, and the upper limits of ∆*z* and ∆*x*_0_ become higher.

The parameters were consistent with the simulation of SFD (Table 1 and Table 2).

## 3. Results

The characteristics and sampling conditions of the algorithms are analyzed and summarized in Table 3, where ∆*x*_0_ is the sampling interval of the initial plane, *N* is the number of horizontal (vertical) sampling points, *L*_0_ is the size of initial plane, *λ* is the wavelength of light, *m* is the scaling factor of the diffraction plane relative to the initial plane, ∆*z* is the diffraction distance, and *r* is the distance between the initial plane and the center of spherical wave for illumination (for a plane wave, *r* tends to +∞).

$m\Delta x_{0}{}^{2}N=\lambda \Delta z$$\Delta x_{0}\leq \frac{\Delta z\lambda }{L_{0}}\sqrt{\frac{r}{\sqrt{2}m(mr-r-\Delta z)}}$Δz≤(m−1)r$\Delta x_{0}\leq \frac{\Delta zr\lambda }{L_{0}(mr-r-\Delta z)}$Δz≤(m−1)r$\Delta x_{0}\leq \frac{\Delta zr\lambda }{L_{0}(mr-r-\Delta z)}$Δz≤(m−1)r It can be seen from the results that for the simulation of large field of view diffraction, three FFT-based algorithms (SFD, SASM, and DBFT) have upper limits on the diffraction distance, ∆*z*, and sampling pitch, ∆*x*_0_. Both ∆*z* and ∆*x*_0_ need to satisfy the sampling conditions to avoid aliasing. If one of them does not satisfy the sampling conditions, a smaller value of the other one will not help. In general, these three algorithms have the same restriction on the diffraction distance, ∆*z*. For the limits on sampling pitch ∆*x*_0_, SASM and DBFT have the same restriction, while SFD has a stricter restriction. Lastly, for the MPASM algorithm based on matrix calculation, we can break through any limits by increasing the parameter *s* to avoid aliasing, but the computational efficiency is generally much lower than other algorithms.

## 4. Discussion

In the simulation of large field of view diffraction, in the case that the centers of the initial plane and diffraction plane are coaxial, SASM and DBFT are more recommended from the aspect of aliasing because of looser sampling restrictions. Before the simulation or after the aliasing occurs during the simulation, the specific parameters can be substituted into the formula in Table 3 to check whether they satisfy the sampling conditions. This paper recommends that the check and adjustment of ∆*z* take precedence over ∆*x*_0_, because the limit on ∆*z* is independent of ∆*x*_0_, while the limit on ∆*x*_0_ is related to ∆*z*. In addition, the restrictions on ∆*z* of various FFT-based algorithms are basically consistent. Therefore, if ∆*z* does not satisfy the sampling conditions and the parameters ∆*z*, *m*, and *r* involved are not adjustable, a smaller value of ∆*x*_0_ will not help; therefore, the FFT-based algorithms should be abandoned, and the MPASM algorithm should be used for calculation to avoid aliasing. However, this generally reduces computational efficiency. If ∆*z* satisfies the condition but there is still aliasing, it means that ∆*x*_0_ does not satisfy the condition and needs to be reduced to below the limit. If it is difficult to reduce ∆*x*_0_, other parameters can be adjusted to increase the upper limit of ∆*x*_0_ according to the specific situation, while ensuring that ∆*z* still satisfies the sampling condition. If both ∆*z* and ∆*x*_0_ satisfy the sampling conditions, there will be no aliasing for FFT-based algorithms.

In addition, since the use of FFT is the source of sampling restrictions, fewer uses of FFT in the algorithm lead to looser sampling restrictions. Therefore, when using FFT-based algorithms, the number of times FFT is used in the algorithm should be paid attention to and minimized. For example, the Fourier transform of some expressions without information of the initial plane can be calculated manually. The algorithm should directly deduce the analytical expression instead of using FFT to calculate the Fourier transform process. Generally, sampling conditions consistent with those of SASM and DBFT can be obtained by reducing the use of FFT to two times. Further use of FFT will make the sampling restrictions stricter.

Lastly, for FFT-based algorithms, this paper summarizes the following problems that are easy to ignore or may be confusing, which can provide reference for the process of analyzing the sampling conditions of other similar algorithms:

1. When analyzing whether the phase distribution in FFT satisfies the Nyquist sampling conditions, sometimes there are two phase factors multiplied. If the two phase factors have similar forms, they should be combined and analyzed to obtain the correct results. If the two phase factors are analyzed separately, the restrictions obtained will be stricter. For example, the phase factor in *U*_1_(*x*_1_, *y*_1_) can be combined with exp[(*ik*/2*z*_2_)(*x*_1_^2^ + *y*_1_^2^)] in Equation (27);

2. When analyzing whether the phase distribution in FFT satisfies the Nyquist sampling conditions, it is necessary to pay attention to its corresponding amplitude distribution synchronously. If there is a blank area in the amplitude distribution, then, even if the phase distribution in this area breaks the Nyquist sampling conditions, it will not affect the results. Accordingly, the sampling restrictions can be further loosened. The process shown in Figure 2 is an example;

3. Sometimes it is necessary to analyze complex phase factors, such as ℱ{*a*(*p*_0_, *q*_0_)} in Equation (9). The theoretical expression of its phase distribution cannot be obtained because *a*(*p*_0_, *q*_0_) in Equation (8) is very complex. However, the factor with less influence in *a*(*p*_0_, *q*_0_) can be ignored to simplify the problem. For example, the influence of the amplitude term and the primary phase term in *a*(*p*_0_, *q*_0_) on the phase distribution of ℱ{*a*(*p*_0_, *q*_0_)} can be explored. If the influence is small, we can ignore them to simplify *a*(*p*_0_, *q*_0_) to a quadratic phase factor, and then calculate the simple approximate expression of ℱ{*a*(*p*_0_, *q*_0_)}.

Lastly, we qualitatively explored the definition of diffraction images at various propagation distances near the focal length at different sampling rates to study the influence of sampling choice on 3D factors. In general, for all propagation distances, including the focal length, when the sampling rate is higher than the original resolution of the initial hologram, the results of different sampling rates do not change. When the sampling rate is lower than the original resolution of the initial hologram, a lower sampling rate will lead to lower diffraction image definition. We plan to go deeper into this issue in another article.

## Figures and Tables

**Table 1:** The Simulation Parameters of Verification 1 of the Sampling Conditions.

Parameters	Values
Wavelength (λ)	632.8 nm
Sampling interval on initial plane ($\Delta x_{0}$)	8 μm
Scaling factor (m)	6
Number of sampling points (N)	1080
Distance from point source to initial plane (r)	150 mm

**Table 2:** The Simulation Parameters of Verification 2 of the Sampling Conditions.

Parameters	Values
Wavelength (λ)	632.8 nm
Propagation distance (Δz)	600 mm
Scaling factor (m)	6
Size of initial plane ($L_{0}=N\Delta x_{0}$)	8.64 mm
Distance from point source to initial plane (r)	150 mm

**Table 3:** Comparison and Summary of Diffraction Algorithms.

Algorithms	Sampling Conditions	Advantages	Disadvantages
S-FFT	$m\Delta x_{0}{}^{2}N=\lambda \Delta z$	Simple calculation	Strict restrictions
SFD	$\Delta x_{0}\leq \frac{\Delta z\lambda }{L_{0}}\sqrt{\frac{r}{\sqrt{2}m(mr\;-\;r\;-\;\Delta z)}}$ Δz≤(m − 1)r	Wide range of use considering that centers of planes are not coaxial	More sampling limits than SASM; requires three FFT operations
SASM	$\Delta x_{0}\leq \frac{\Delta zr\lambda }{L_{0}(mr\;-\;r\;-\;\Delta z)}$ Δz≤(m − 1)r	Easy to implement with two FFT operations	Diffraction distance cannot be too large
DBFT	$\Delta x_{0}\leq \frac{\Delta zr\lambda }{L_{0}(mr\;-\;r\;-\;\Delta z)}$ Δz≤(m − 1)r	Simple principle with two FFT operations	Diffraction distance cannot be too large
MPASM	No limit with parameters adjusted	Wide range of use; sampling limit can be broken by adjusting parameters	High computational complexity

## Data Availability

Not applicable.
